# The effect of chemotherapy combined with recombination mutant human tumor necrosis factor on advanced cancer

**DOI:** 10.1186/1479-5876-2-33

**Published:** 2004-10-14

**Authors:** Xing Liu, Xiang-fu Zhang, Zhi-weng Zheng, Huishan Lu, Xinyuan Wu, Changmin Huang, Chuan Wang, Guoxiang Guang

**Affiliations:** 1 Department of Oncology, Union Hospital of Fujian Medical University, Fujian, Fuzhou, 350001, P. R. China

**Keywords:** Tumor, Tumor necrosis factor, Biological therapy, Chemotherapy, Complex therapy

## Abstract

**Background:**

Past studies suggested that tumor necrosis factor (TNF) assisted anti-tumor treatment and intensified the sensitivity of chemotherapy. However its clinical application has been curbed because of its low purity, high dosage, and strong toxicity. This research, through perspective random clinical control experiment, observed the therapeutic effect of the treatment of late malignant tumor through the injection of recombinant mutant human tumor necrosis factor (rmhTNF) combined with general chemotherapy and its adverse reactions.

**Methods:**

105 patients with advanced malignant tumor were randomly divided into trial group, 69 patients, and control group, 36 patients. Injection of rmhTNF 4 × 10^6^u/m^2 ^was given to the trial group, from the 1^st ^to 7^th ^days, the 11^th ^to 17^th ^days combined with chemotherapy course. The chemotherapy plan was as follows: CAP for patients with the NSCLC; FAM for patients with gastric cancer; FC for patients with colorectal cancer. One treatment cycle lasted for 21 days and two cycles were scheduled. The control group was given only the same chemotherapy as the trial group.

**Results:**

In the trial group there was 1 CR case and 12 PR cases, and the response rate is 13/69 (18.84%); in the control group 1 PR case, the response rate 1/36 (2.78%). The response rate of the trial group was significantly higher than that of the control group (*P *= 0.022). The response rate for NSCLC in the trial group was 8/17 (47.06%), and 1/6 (16.67%) in the control group. The response rates for gastric cancer and colorectal cancer in the trial groups also were higher than those of the control groups. After the treatment the KPS is 89.00 ± 9.92 in the trial group, and 84.17 ± 8.84 in the control group, with a significant difference between the two groups (*P *= 0.028). The adverse reactions of rmhTNF injection included: pain in the injection area, chill, hardening and swelling and redness in the injection area, fever, ostealgia and myosalgia, and cold-like symptoms. All these adverse reactions were mild and bearable.

**Conclusions:**

The administration of rmhTNF injection in combination with general chemotherapy is an effective and secure means in treating advanced malignant tumor.

## 

Tumor necrosis factor (TNF) is a polypeptide produced by monocytic macrophages and T-lymphocytes stimulated by endotoxin[1]. It has been well shown in vivo or in vitro that TNF assists anti-tumor treatment and intensified the sensitivity of chemotherapy to many different kinds of tumor cells [2-5]. However its clinical application has been curbed because of its low purity, high dosage, and strong toxicity. Via the cooperation between the Forth Military Medical University and Shanghai Celstar Bio-pharmaceutical Holding Co. Ltd, an injection of recombinant mutant human tumor necrosis factor (rmhTNF) which is a genetic engineering TNF of a high activity and low toxicity, has been produced by rebuilding natural TNF with protein engineering technics. Phase I clinical trial (tolerance test) showed that the patients had good tolerance. and the toxicity of rmhTNF was slight As a participant of the Phase II and Phase III clinical study of rmhTNF, we observed the therapeutic effect of the treatment of late malignant tumor through the injection of recombinant mutant human tumor necrosis factor combined with general chemotherapy and its toxicity in a multi-center, random clinical control Phase II and Phase III clinical study of rmhTNF during October 2000 ~ May 2002. The results are reported as below.

## 1. Materials and Methods

### 1.1 Patients

105 patients from our department with advanced malignant tumors diagnosed via pathologic or cytological examinations were randomly collected, of which 23 were non-small cell lung cancer, 50 gastric cancers and 32 were colorectal cancers. Moreover, 79 of them were males and 26 of them were females. The range of their ages was 25~70, with the median age 52 years old. All of them were during their later phases of either recrudescent or metastatic cancers, had no sugary indexes and were taking conservative medicine treatment. KPS scoring for them before treatment were ≥ 60, neutrophil ≥ 2.0 × 109/L, PLT ≥ 100 × 109/L and Hb ≥ 90 g/L. They had almost normal functions of the heart, liver and kidney and at least had one evaluable tumor focus. They had not taken any other anti-tumor treatment one month before the experiment and they had a prospective survival time > 3 months. All of the patients were well informed and written consents were signed. Then they were randomly divided into two groups, i.e. the trial group of 69 patients and the control group of 36 patients, the conditions of the two groups were comparable (*P *>0.05, see Table 1). In the individual group of three tumors, the stage of the disease is of no significant difference (*P *>0.05, see Table 1).

**Table 1:** The General Data of the Trial and Control Group

Item		Trial group (n = 69)	Control group (n = 36)	*P*
Sex				0.362
Male		50	29	
Female		19	7	
Median age (year)		52	51	0.437
Type of tumors				0.638*
NSCLC		17	6	
Gastric cancer		32	18	
Colorectal cancer		20	12	
KPS ()		85.31 ± 6.07	86.69 ± 7.81	0.334
Clinical stage				0.630
III		11	8	
IV		58	28	
NSCLC	III	5	3	0.621*
	IV	12	3	
Gastric cancer	III	4	3	0.684
	IV	28	15	
Colorectal cancer	III	2	2	0.620*
	IV	18	10	

*The *p *value is calculated by Fisher exact chi-square because n < 30 or some cells have expected count less than 1.

### 1.2 Source of drugs

Injection of rmhTNF which was in powder form of 500 × 10^5^u/tube (number 00061) was made and provided by the Forth Military Medical University and was stored at 4°C in refrigerator.

### 1.3 Treatment protocol

For the trial group, injection of rmhTNF 4 × 10^6^u/m^2 ^was given from the 1^st ^to 7^th ^days, the 11^th ^to 17^th ^days combined with chemotherapy course. The chemotherapy plan was as follows: CAP (CTX 750 mg/m^2^, d_1_, ADM 40 mg m^2^, d_1_, DDP 30 mg m^2^, d_1_-d_3_) for patients with the non-small cell lung cancer; FAM (5-FU500 mg/ m^2^, d_1_-d_5_, ADM 40 mg/ m^2^, d_1_, MMC 6 mg/ m^2^, d_1_) for patients with gastric cancer; FC (5-FU 500 mg/ m^2^, d_1_-d_5_, CF 100 mg/ m^2^, d_1_-d_5_) for patients with colorectal cancer. One treatment cycle lasted for 21 days and two cycles were scheduled. For the control group, only the same chemotherapy as the trial group was given. Signs, symptoms and adverse reactions were carefully observed during the treatment. Weekly examinations of blood routine were performed before and after treatment, while liver and kidney functions, urine routine, EEG, liver ultrasonic and chest X-ray examinations were performed before and after every treatment cycle. CT examinations of the evaluable tumor focuses were performed one time before treatment, when treatments were finished and after 4 weeks of the finish.

### 1.4 Evaluation of response and toxicity

#### 1.4.1 Evaluation of response

Complete response (CR) is that, the disappearance of all lesions and no appearance of new disease for at least 4 weeks. Partial response (PR) is defined as a reduction by at least 50% in the sum of the products of the two longest diameters of all lesions maintained for at least 4 weeks with no appearance of new disease. Minimal response (MR) is different from PR with a reduction by at least 25%, but not more than 50%. Stable disease (SD) is a less than 25% reduction or less than 25% increase in the sum of the products of the two perpendicular diameters of all measured lesion with no appearance of new disease. Progression disease (PD) is that, an increase greater than 25% over the size present at entry into the study or, for patients who respond, the size at time of maximum regression, or the appearance of new areas of malignant disease. CR+PR were rated as response rate.

#### 1.4.2 Toxicity

The toxicity was followed the WHO acute and sub-acute toxic rating

## 2. Results

### 2.1 Response to treatment

After two treatment cycles, the trial group had 1 CR case, 12 PR cases, 11 SD cases and 18 PD cases, and the response rate was 13/69(18.84%). The control group, on the other hand, had 1 PR case, 4 MR cases, 19 SD cases and 12 PD cases, and had a response rate 1/36 (2.78%). The response rate of the trial group was significantly higher than that of the control group (*P *= 0.022, see Table 2).

**Table 2:** The Response Rate in the Trial and Control Group after Two Treatment Cycles

Group	n	CR	PR	MR	SD	PD	Response rate	*P*
Trial	69	1	12	11	27	18	13/69 (18.84%)	0.022
Control	36	0	1	4	19	12	1/36 (2.78%)	

The response rate for NSCLC of the trial group was 8/17 (47.06%), higher than that of the control group 1/6 (16.67%) but without statistic significance (*P *= 0.208). The response rates for gastric cancer was 12.50% (4/32), while for the controls were 0.00% (0/18) without statistic significance (*P *= 0.283). The response rates for colorectal cancer was 5.00% (1/20), while no response case in the controls group (0/12), but there was still no statistic significance (*P *= 1.000, see Table 3).

**Table 3:** The Response Rate in the Trial and Control Group for Different Type of Tumors

	Group	n	CR	PR	MR	SD	PD	Response rate	p*
NSCLC	Trial	17	1	7	3	4	2	47.06% (8/17)	0.208
	Control	6	0	1	1	3	1	16.67% (1/6)	
Gastric cancer	Trial	32	0	4	6	13	9	12.50% (4/32)	0.283
	Control	18	0	0	2	9	7	0.00 (0/18)	
Colorectal cancer	Trial	20	0	1	2	10	7	5.00% (1/20)	1.000
	Control	12	0	0	1	7	4	0.00 (0/12)	

*The *p *value is calculated by Fisher exact chi-square because n < 30 or some cells have expected count less than 1.

### 2.2 Life quality

Before the treatment there was no significant difference of the general status (KPS) between the two groups (*P *> 0.05). After the treatment the KPS was 89.00 ± 9.92 in the trial group, and 84.17 ± 8.84 in the control group, with a statistic significant difference (p = 0.028, see Table 4).

**Table 4:** Before and after Treatment, KPS Value in the Trial and Control Group

Group	Trial (n = 69)	Control (n = 36)	*P*
Before treatment ()	85.31 ± 6.07	86.69 ± 7.81	0.334
After treatment ()	89.00 ± 9.92	84.17 ± 8.84	0.028

### 2.3 Toxicity

Fever, cold-like symptoms, ostealgia and myosalgia, chill, pain in the injection area, hardening and swelling and redness in the injection area were much more happened in the trail group compared with the control group (P < 0.01 = , while there were no significant differences between the two groups on the frequencies of anemia, leukopenia, thrombocytopia and nausea / vomiting (P > 0.05). No abnormalities correlated with drugs were found in liver or kidney functions, urine routine, EEG and blood pressure (see Table 5).

**Table 5:** Toxicity in the Trial and Control Group

Toxicity	Trial (n = 69)					Control (n = 36)					P*
			
	0	I	II	III	IV	0	I	II	III	IV	
Anemia	37	16	8	7	1	21	9	4	1	1	0.665
Leukopenia	28	23	9	9	0	17	13	4	2	0	0.570
Thrombocytopia	59	5	2	2	1	31	3	1	1	0	0.557
Nausea / Vomiting	32	16	19	2	0	18	4	11	2	1	0.476
Fever	52	12	5	0	0	36	0	0	0	0	0.000
Eruption	67	2	0	0	0	36	0	0	0	0	0.274
Cold-like symptoms	47	19	3	0	0	36	0	0	0	0	0.000
Ostealgia / Myosalgia	46	21	2	0	0	36	0	0	0	0	0.000
Chill	38	31	0	0	0	36	0	0	0	0	0.000
Pain in injection area	13	41	15	0	0	36	0	0	0	0	0.000
Hardening, swelling and redness in injection area	42	17	10	0	0	36	0	0	0	0	0.000

*The *p *value is calculated by Fisher exact chi-square because n<30 or some cells have expected count less than 1.

## 3. Case report

**Figure 1:**
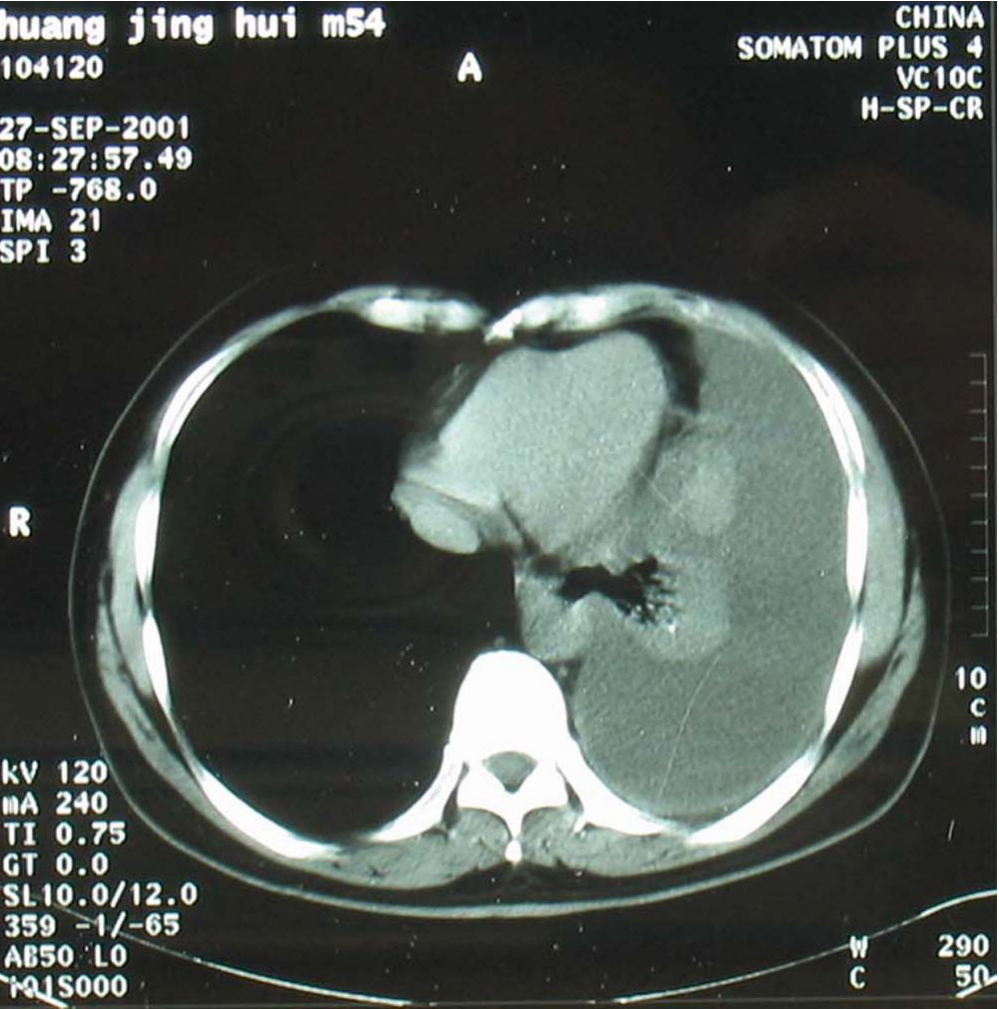
Chest CT before treatment (27-Sep-2001) show that conglomeration of a size of 5.5 × 4.2 cm at the left lower hilus pulmonis, large amount of accumulation of fluid in the left thoracic cavity, enlarged lymph nodes in the mediastinum.

**Figure 2:**
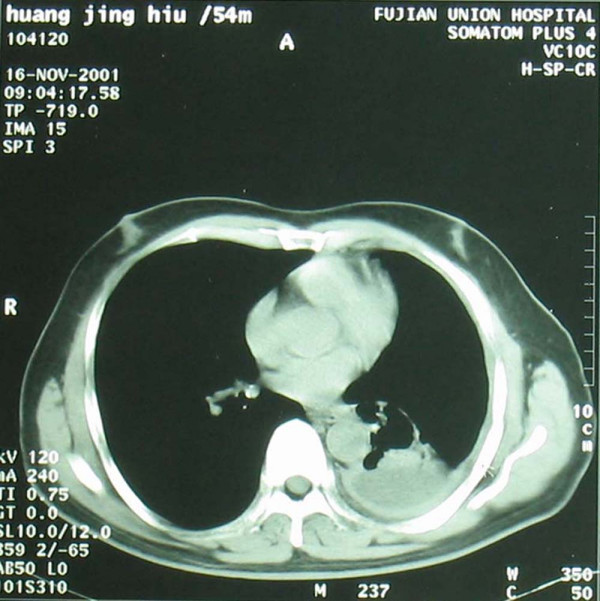
Chest CT after treatment (16-Nov-2001) show that clear shrinkage of conglomeration of 3.0 × 2.5 cm at the left lower hilus pulmonis, small amount of accumulation of fluid in the left thoracic cavity.

A 54-year-old man hospitalized at August 27, 2001 with complains of "left chest pain accompanied by cough and hard breath for half a month". Physical examination after hospitalization showed: enlarged lymph node of 3 × 3 cm above the right clavicle, hard and immobile. Chest CT on September 27, 2001 (see Figure 1) showed: conglomeration of a size of 5.5 × 4.2 cm at the left lower hilus pulmonis, large amount of accumulation of fluid in the left thoracic cavity, enlarged lymph nodes in the mediastinum. Biopsy of the lymph node above the right clavicle showed: transferred adenocarcinoma. Cancer cells were found in the fluid in the thoracic cavity after centrifugation. The diagnosis was "Adenocarcinoma on the left lower lung, stage T_4_N_3_M_0_IIIb". Chemotherapy of protocol CAP + rmhT NF injection (i.m.) was given from October 4 to November 14, 2001. Two weeks later, a clear relief of hard breath and cough was found. After two periods of therapy (November 16, 2001), physical examination showed shrinkage of lymph node of 0.5 × 0.5 cm above the right clavicle, and Chest CT (see Figure 2) showed: clear shrinkage of conglomeration of 3.0 × 2.5 cm at the left lower hilus pulmonis, small amount of accumulation of fluid in the left thoracic cavity. A callback of CT one month later showed: conglomeration was of the size of 4.0 × 2.8 cm. The curative effect was confirmed as "PR".

## 4. Discussion

Tumor necrosis factor (TNF) is a polypeptide produced by monocytic macrophages and T-lymphocytes stimulated by endotoxin. It can act as modulator to immunity and induces anti-tumor effects in hosts. It also has direct cytotoxic effects and inhibitory effects on cellular growth. It can kill the tumor cell without notable toxic effect on the normal cells [1]. Studies have shown that the anti-tumor mechanism of the tumor necrosis factor includes 1) killing the tumor cell directly [3]; 2 inducing the apoptosis of tumor cells [3]; 3) reversing more drug resistance of tumor cell and improving the sensitiveness of chemotherapy [3]; 4) destroying the blood supply of tumor tissue [4]; 5) increasing the killing effects of immune-effect cells on the tumor cells [3]. However, its clinical application has been curbed because of its low purity, high dosage, and strong toxicity. Studies have also shown that, a higher anti-tumor effect and lower toxicity were got by modified some structure of TNF. Nakamura [6] prepared a novel recombinant tumor necrosis factor-α (TNF) mutant (mutant 471), in which 7 N-terminal amino-acids were deleted and Pro^8^Ser^9^Asp^10 ^was replaced by Arg-Lys-Arg, and compared its biological activity with that of wild-type recombinant TNF. Mutant 471 had a 7-fold higher anti-tumor activity against murine L-M cells in vitro, and a higher binding activity to TNF receptors on L-M cells, than wild-type TNF. Kamijo[7] reported that TNF(C-Phe), in which the C-terminal leucine of TNF molecule was replaced by phenylalanine, was 20-times as potent in induction of differentiation of human myelogenous leukemia cells (U-937 cells) as the parent TNF(N-Met). The rmhTNF has been obtained successfully from hTNF-α by gene engineering technology which was mutated by deleting 7 amino acids at the N-terminus, replacing Pro^8^Ser^9^Asp^10 ^by Arg-Lys-Arg, and substituting Leu157 with Phe [8]. Phrase I clinical trial indicated that, all of 32 patients who were randomly grouped into six groups were treated with different dose: 2.5 × 10^5^u/m^2^, 5 × 10^5^u/m^2^, 1 × 10^6^u/m^2^, 2 × 10^6^u/m^2^, 3 × 10^6^u/m^2^, 4 × 10^6^u/m^2^, were well tolerable.

The present study has shown that shortly after the combined chemotherapy with rmhTNF, 24/69 (34.78%) of the focuses were more or less absorbed or subsidized, of which there were 1 CR case and 12 PR cases, with the response rate was 13/69 (18.84%), while in the control group only 5/36 (13.89%) cases had reduced focuses with only 1 PR case and the response rate 1/36 (2.78%). The response rate of the trial group was significantly higher than that of the control group (*P *= 0.022). Analysis of the therapeutic effects in different kind of diseases showed that the response rate for NSCLC of the trial group was 8/17 (47.06%), higher than that of the control group 1/6 (16.67%), which also means that TNFα has suppressive effects on lung adenocarcinoma[9]. The reason why there was no statistic significance (*P *> 0.05) between the two groups may be due to too small samples in this investigation. The response rates for gastric cancer and the colorectal cancer were both quite low, i.e. 4/32 (12.50%) and 1/20 (5.00%), respectively, while for the controls were 0/18 and 0/12, respectively (0.00%) and without statistic significance (*P *> 0.05). Such a result may be mainly due to the fact that most of the patients had taken combined chemotherapy before and that samples in both groups were quite small. It was thought [2] that the anti-tumor activity of TNF may co-activate some chemotherapy drugs. Results of the present study showed that the combined therapy of TNF and chemotherapy may be still effective on previously chemotherapy drug resistant tumors, which also means that TNF has co-activating effects on chemotherapy drugs. Moreover, after two treatment cycles, scoring for the general status (KPS) was 89.00 ± 9.92 in the trial group and 84.17 ± 8.84 in the control group, with a statistic significant difference (*P *= 0.028). It shows that the combined rmhTNF chemotherapy may benefit the quality of the patients' life.

It has already been reported[2-5,10,11] that the toxicity of TNF in phase I or II clinical trials are mainly chill, fever, local redness, swelling and pain, hypotension, nausea, vomiting, myalgia, fatigue and diarrhea, while pulmonary hemorrhage and severe hepatic dysfunction also have been observed[12]. But there are no reports on the toxicity of rmhTNF. The present study showed that some of the patients had pain in the injection area, chill and hardening, swelling and redness in the injection area of which the incidences were 81.2%(56/69), 44.9%(31/69) and 39.1% (27/69), respectively, but of a low degree and the patients had good tolerance. The symptoms of fever, ostealgia and myosalgia and cold-like symptoms were more in the trail group than that in the control group, all of which were of grade I or II. They mainly happened when the drugs were given at the 3^rd ^or 5^th ^times, and can be relieved after taking 25 mg metacen, and can disappear automatically after the finish of the therapy. There were no significant differences between the two groups on the number of cases which showed marrow suppression or nausea / vomiting, and on the degree of those symptoms. There were no cases which showed rmhTNF-correlated abnormal liver or kidney functions, urine routine, EEG and blood pressure.

In summary, combined therapy of rmhTNF and chemotherapy has co-activating and sensitivity improving effects on the treatment of advanced malignant tumors, and may increase the recent responsive effectiveness with an improvement of the general status and quality of patients' lives. The main adverse reactions of the local injection of rmhTNF are pain in the local injection area, chill, hardening and swelling and redness in the injection area, fever, ostealgia and myosalgia and cold-like symptoms, all of which were of light to moderate degree and are tolerable.
